# A Novel Hemizygous *ANOS1* Variant in a Patient With Kallmann Syndrome and Type 2 Diabetes Mellitus: A Case Report

**DOI:** 10.1155/crie/8624241

**Published:** 2026-07-22

**Authors:** Si Qin, Yindi Zhang, Yanrong Chen, Wei Chen, Yinxing Ni, Jian Zhong

**Affiliations:** ^1^ Department of Endocrinology, The Third Affiliated Hospital of Chongqing Medical University, Chongqing, China, cqmu.edu.cn; ^2^ Department of Radiology, The Third Affiliated Hospital of Chongqing Medical University, Chongqing, China, cqmu.edu.cn

**Keywords:** *ANOS1*, case report, congenital hypogonadotropic hypogonadism, Kallmann syndrome, type 2 diabetes mellitus

## Abstract

**Introduction:**

Kallmann syndrome (KS), a rare genetic disorder, is characterized by congenital hypogonadotropic hypogonadism (CHH) and anosmia or hyposmia.

**Case Report:**

We report a case of KS with type 2 diabetes mellitus (T2DM) associated with a novel hemizygous missense variant in *ANOS1* (NM_000216.4; exon 4; c.505G >C;p.(Gly169Arg)). A 33‐year‐old male presented with persistent hyperglycemia, delayed pubertal development with absent secondary sexual characteristics, and lifelong anosmia. Olfactory testing, magnetic resonance imaging (MRI) of the pituitary region, and a gonadotropin‐releasing hormone (GnRH) stimulation test supported a clinical diagnosis of KS. Trio‐based exome sequencing (ES) revealed the aforementioned hemizygous variant in *ANOS1*. Notably, neither parent harbored this variant, supporting a de novo origin, which is consistent with the variant’s potential pathogenicity. Multiple sequence alignment showed that Gly169 is highly conserved across species, suggesting it may be functionally important for the protein. Furthermore, a three‐dimensional protein model generated using SWISS‐MODEL predicted marked conformational changes in the protein attributable to this amino acid substitution at a highly conserved residue, suggesting a potential functional impact. Notably, emerging evidence has linked *ANOS1* variants to dysregulated glucose metabolism, supporting a potential association with T2DM in this case. For treatment, insulin and oral hypoglycemic agents were used for glycemic control, and a GnRH pump was initiated. At the sixth follow‐up visit, the patient had achieved satisfactory glycemic control; however, serum testosterone levels remained low despite GnRH pump therapy, so testosterone replacement therapy was subsequently initiated.

**Conclusions:**

*ANOS1* variants are genetically diverse and may contribute to both KS pathogenesis and the development of comorbid T2DM, though further studies are needed to confirm this link. For KS patients, close monitoring of fasting glucose, glycated hemoglobin, and oral glucose tolerance test results is advisable to detect diabetes and prediabetes early.

## 1. Introduction

Kallmann syndrome (KS) (Online Mendelian Inheritance in Man: 308,700), a rare genetic disorder, is characterized by congenital hypogonadotropic hypogonadism (CHH) with concurrent anosmia or hyposmia [1, 2]. Epidemiological data indicate that KS affects approximately 1 in 8000 males, with a prevalence about fivefold lower in females [3]. Two‐thirds of cases are familial, while the remaining cases are sporadic [4]. The pathogenesis of KS is incompletely understood but is thought to involve impaired development of gonadotropin‐releasing hormone (GnRH) neurons, leading to GnRH deficiency, alongside olfactory dysfunction [5]. GnRH neurons are unique neuroendocrine cells that develop in the olfactory placode and migrate to the forebrain during embryonic development. GnRH neurons navigate to the forebrain by following the axonal pathways that extend from olfactory neurons toward the olfactory bulb [6]. This migration is critical for GnRH neurons to integrate into the hypothalamic–pituitary axis. Consequently, the absence of olfactory bulbs results in GnRH neurons becoming isolated from the central nervous system [6]. KS arises from pathogenic variants in over 30 genes, most commonly *ANOS1*, *FGFR1*, and *PROK2* [7–9], with inheritance patterns including autosomal recessive, autosomal dominant, and X‐linked recessive [5].

Currently, diabetes mellitus (DM) is classified into type 1 DM, type 2 DM (T2DM), gestational DM, and other specific types, with T2DM accounting for approximately 90% of global cases [10]. Moreover, chronic sex hormone deficiency, a hallmark of KS, is thought to contribute to various metabolic disturbances, including hypertension, osteoporosis, dyslipidemia, insulin resistance, and potentially DM [11]. Recent research has reported an *ANOS1* variant (p.Cys172Phe) in KS patients with DM [11]. However, the hemizygous *ANOS1* variant c.505G >C;p.(Gly169Arg) and its potential association with both KS and DM have not been previously reported.

This case report describes a novel *ANOS1* variant identified in a 33‐year‐old male patient with both KS and T2DM, which may enhance our understanding of the genetic and metabolic mechanisms underlying this syndrome.

## 2. Materials and Methods

Peripheral blood‐derived DNA from the proband and his parents was subjected to exome sequencing (ES) to identify potential pathogenic variants. The Human All Exon GenCap capture kit (MyGenostics Inc., Beijing, China) was used for hybrid selection. Sentieon software (v202112.06, under default parameters) was used to identify single‐nucleotide polymorphisms, insertions, and deletions. Sequencing data were converted to a variant call format to standardize subsequent analyses. Single nucleotide variants were filtered to retain only exonic and splice‐site variants, with a read depth ≥ 100 × genotype quality ≥ 20. Variant annotation was performed using ANNOVAR software (version 2020‐06‐11, http://annovar.openbioinformatics.org/en/latest/) based on the Genome Reference Consortium Human Build 38 (GRCh38/hg38) reference genome, incorporating multiple databases including 1000 Genomes Project, Database of Single Nucleotide Polymorphisms (dbSNPs), MyGenostics in‐house database, Human Gene Mutation Database (HGMD), ES Project 6500 (ESP6500), and Exome Aggregation Consortium (ExAC). Population frequencies were annotated using the 1000 Genomes Project (1000g2015aug_all) and Genome Aggregation Database (gnomAD) exome databases (gnomAD_exome_ALL and gnomAD_exome_East Asian), and variants with a minor allele frequency >0.01 were excluded. Subsequently, the functional impact of these variants was predicted using a suite of algorithms: Sorting Intolerant From Tolerant (SIFT), MutationTaster, Polymorphism Phenotyping v2 (PolyPhen‐2), Genomic Evolutionary Rate Profiling++ (GERP++), and Rare Exome Variant Ensemble Learner (REVEL) (Supporting Information 1: Table S1). The variant and corresponding phenotypic data have been submitted to ClinVar (submission ID: SUB16267931, https://www.ncbi.nlm.nih.gov/clinvar/).

The sequence of the human ANOS1 protein was retrieved from the HomoloGene database (National Center for Biotechnology Information), and Unipro UGENE (version 33.0) [12] was used to assess the conservation of amino acid residues in the relevant peptide regions. For ANOS1 structural predictions, the online SWISS‐MODEL platform (https://swissmodel.expasy.org/interactive) was used to predict the three‐dimensional structure of the ANOS1 protein. Additionally, PyMOL 2.3.0 was used for structural visualization.

### 2.1. Sanger Sequencing

All candidate variants identified by the Illumina NovaSeq 6000 sequencer were further validated via Sanger sequencing. Genomic DNA was extracted from all available family members. One pair of primers was designed to amplify the target region of *ANOS1* (forward: 5′‐AATGACTTGCTCTGCCCCAT‐3′ and reverse: 5′‐CCTGGGGTGACAAAGTGAGA‐3′). Polymerase chain reaction (PCR) amplification was performed under the following conditions: initial denaturation at 96°C for 3 min; 7 cycles of denaturation at 96°C for 15 s, annealing at 66°C for 30 s, and extension at 72°C for 45 s; 10 cycles of denaturation at 95°C for 15 s, annealing at 62°C for 30 s, and extension at 72°C for 45 s; another 10 cycles of denaturation at 95°C for 15 s, annealing at 58°C for 30 s, and extension at 72°C for 45 s; eight cycles of denaturation at 95°C for 15 s, annealing at 54°C for 30 s, and extension at 72°C for 45 s; a final extension at 72°C for 5 min; and a final hold at 4°C. PCR products were purified via standard sodium acetate–ethanol precipitation. The purified PCR products were subjected to Sanger sequencing on an ABI 3730XL (Applied Biosystems, USA). The sequencing results were analyzed using Mutation Surveyor v5.1.2 (SoftGenetics, State College, PA, USA).

## 3. Case Presentation

A 33‐year‐old man was referred on May 17, 2024, for the evaluation of newly identified hyperglycemia. The patient presented with normal height but exhibited arrested secondary sexual development. His penile hypoplasia and bilateral testicular hypoplasia were noted on the clinical examination. Notably, he lacked secondary sexual characteristics, including the absence of a distinct laryngeal prominence and failure of voice masculinization. Axillary, facial, and pubic hair growth was absent, and the patient denied morning erections or nocturnal emissions. Of critical clinical significance, he reported lifelong congenital anosmia. Cognitive development was on par with his chronological age, and his academic performance was average for the peer group. He was born at term; however, no birth measurements (e.g., length and weight) were documented. His parents were non‐consanguineous, and there was no family history of genetic disease or pubertal delay. However, both his mother and maternal grandmother suffered from T2DM. His height was 171 cm, and his weight was 68 kg, with a body mass index of 23.3 kg/m^2^. Both height and weight fell between the 25th and 50th percentiles for age‐ and sex‐matched Chinese individuals [13].

**Figure 1:**
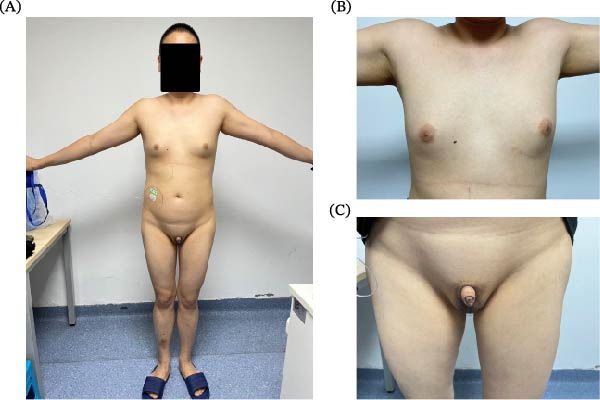
Physical examination. (A) Absent axillary hair and pubic hair in the patient. (B) Bilateral gynecomastia. (C) Bilateral underdevelopment of the external genitalia.

On physical examination, the patient exhibited prepubertal scrotal characteristics, with non‐palpable bilateral testes (Figure 1A,C). Facial hair was absent, including the mustache area; the laryngeal prominence was underdeveloped, and the voice was high‐pitched and soft. Axillary, pubic, and leg hair was sparse. In addition to overt gynecomastia, a firm glandular tissue was palpable beneath the bilateral areolae (Figure 1B). Tanner staging was G1 for genitalia and P2 for pubic hair.

Notably, hormonal analysis revealed markedly low serum testosterone levels and reduced concentrations of luteinizing hormone (LH) and follicle‐stimulating hormone (FSH) (Table 1). The GnRH stimulation test and the human chorionic gonadotropin (hCG) stimulation test both supported the diagnosis of hypogonadotropic hypogonadism consistent with KS (Tables 2 and 3). According to protocols reported in previous olfactory studies [14], olfactory function was severely impaired as the patient was unable to distinguish the odors of vinegar and alcohol (with water as a negative control) during olfactory testing.

**Table 1:** Auxological parameters and hormone levels of the patient.

Parameter	Value	Units	Laboratory reference ranges	Assay platforms
Age	33	years	—	—
Height	171	cm	—	—
Weight	68	kg	—	—
Body mass index	23.3	kg/m^2^	—	—
Father’s height	160	cm	—	—
Mother’s height	160	cm	—	—
Predicted adult height	165	cm	—	—
Free thyroxin	13.92	pmol/L	7.98–16.02	Chemiluminescent immunoassay, ROCHE cobas e801
Free triiodothyronine	4.62	pmol/L	3.53–7.37	Chemiluminescent immunoassay, ROCHE cobas e801
Thyroid‐stimulating hormone	0.552	μIU/mL	0.56–5.91	Chemiluminescent immunoassay, ROCHE cobas e801
Testosterone	1.21	nmol/L	8.64–29.00	Chemiluminescent immunoassay, ROCHE cobas e801
Estradiol	<15	pg/mL	<15–38.95	Chemiluminescent immunoassay, ROCHE cobas e801
Progesterone	<0.1	ng/mL	0.14–2.06	Chemiluminescent immunoassay, ROCHE cobas e801
Follicle‐stimulating hormone	0.86	mlU/mL	1.27–19.26	Chemiluminescent immunoassay, ROCHE cobas e801
Luteinizing hormone	0.38	mlU/mL	1.24–8.62	Chemiluminescent immunoassay, ROCHE cobas e801
Prolactin	5.09	ng/mL	2.64–13.13	Chemiluminescent immunoassay, ROCHE cobas e801
Dehydroepiandrosterone sulfate	142	μg/dL	106–464	Chemiluminescent immunoassay, ROCHE cobas e801
17 alpha‐hydroxyprogesterone caproate	0.21	ng/mL	0.31–2.01	Liquid chromatography‐tandem mass spectrometry (AB SCIEX API 3200MD; SCIEX, Concord, ON, Canada)
Androstenedione	1.13	nmmol/L	1.4–6.63	Liquid chromatography‐tandem mass spectrometry (AB SCIEX API 3200MD; SCIEX, Concord, ON, Canada)
Adrenocorticotropic hormone at 8:00 A.M	13.8	pg/mL	7.2–63.3	Chemiluminescent immunoassay, ROCHE cobas e801
Blood plasma cortisol at 8:00A.M	8.29	μg/dL	AM: 6.71–22.54	Chemiluminescent immunoassay, ROCHE cobas e801
Growth hormone level	0.22	ng/mL	0–0.97	Chemiluminescent immunoassay, ROCHE cobas e801
Insulin‐like growth factor‐1	185	μg/L	71–234	Chemiluminescent immunoassay, ROCHE cobas e801
Fasting plasma glucose	14.74	mmol/L	3.9–6.1	Hexokinase method, ROCHE cobas c701
Glycated hemoglobin	12.3%	%	4–6.2	High‐performance liquid chromatography, Premier Hb9210

**Table 2:** Gonadotropin‐releasing hormone stimulation test.

Time (min)	LH	Units	Laboratory reference ranges	Assay platforms	FSH	Units	Laboratory reference ranges	Assay platforms
0	0.28	mlU/mL	1.24–8.62	Chemiluminescent immunoassay, ROCHE cobas e801	0.78	mlU/mL	1.27–19.26	Chemiluminescent immunoassay, ROCHE cobas e801
30	2.05	mlU/mL	—	—	1.79	mlU/mL	—	—
60	2.57	mlU/mL	—	—	2.67	mlU/mL	—	—
90	2.57	mlU/mL	—	—	2.8	mlU/mL	—	—
120	2.52	mlU/mL	—	—	2.94	mlU/mL	—	—

**Table 3:** Human chorionic gonadotropin stimulation test.

Time (h)	Testosterone	Units	Laboratory reference ranges	Assay platforms
Baseline	0.35	ng/mL	8.64–29.00	Chemiluminescent immunoassay, ROCHE cobas e801
24	0.24	ng/mL	—	—
48	0.22	ng/mL	—	—
72	0.34	ng/mL	—	—

**Figure 2:**
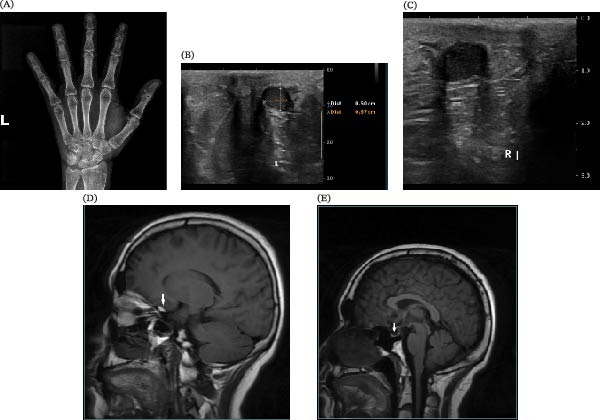
Bone age assessment, ultrasound, and magnetic resonance imaging (MRI) findings for the proband. (A) Digital radiography revealed complete epiphyseal closure. (B) Ultrasound imaging of the left testis revealed dimensions of 7 mm × 7 mm × 13 mm. (C) Ultrasound imaging of the right testis revealed dimensions of 6 mm × 7 mm × 12 mm. (D) The coronal brain MRI report demonstrated olfactory bulbs, revealed by white arrows. (E) Contrast‐enhanced MRI of the pituitary gland revealed a partially empty sella, as denoted by white arrows.

Bone age radiography revealed complete epiphyseal closure (Figure 2A). Scrotal ultrasonography revealed markedly reduced testicular volumes (right: ~2.6 mL; left: ~3.1 mL, calculated as length × width × thickness × 0.52), as shown in Figure 2B,C. Brain and pituitary magnetic resonance imaging (MRI) revealed structurally intact olfactory bulbs and a partially empty sella turcica, as visualized in Figure 2D,E. Previous studies have demonstrated that normal olfactory function can be preserved in all six patients with unilateral olfactory bulb aplasia and three patients with bilateral aplasia [15]. Therefore, normal function does not necessarily depend on structural integrity, and an intact structure does not ensure normal function. In the present case, although the olfactory bulbs were structurally intact on imaging, normal olfactory function was not preserved.

DM was confirmed by elevated fasting serum glucose, increased glycated hemoglobin (HbA1c), and abnormal oral glucose tolerance test results, as detailed in Tables 1 and 4. The glucose‐stimulated C‐peptide secretion test indicated no absolute C‐peptide deficiency. Moreover, islet cell antibodies, insulin autoantibodies, and glutamic acid decarboxylase antibodies were negative. These findings are consistent with the diagnosis of T2DM. Notably, the absence of islet autoantibodies and preserved C‐peptide secretion, alongside a family history of T2DM, further support this diagnosis.

**Table 4:** Plasma glucose and C‐peptide responses to oral glucose tolerance test.

Time (min)	Plasma glucose	Units	Laboratory reference ranges	Assay platforms	C‐peptide	Units	Laboratory reference ranges	Assay platforms
0	6.54	mmol/L	3.9–6.1	Hexokinase method, ROCHE cobas c701	1.76	ng/mL	1.1–4.4	Chemiluminescent immunoassay, ROCHE cobas e801
30	11.24	mmol/L	—	—	2.85	ng/mL	—	—
60	14.64	mmol/L	—	—	3.44	ng/mL	—	—
90	15.89	mmol/L	—	—	4.38	ng/mL	—	—
120	13.74	mmol/L	—	—	4.01	ng/mL	—	—

**Figure 3:**
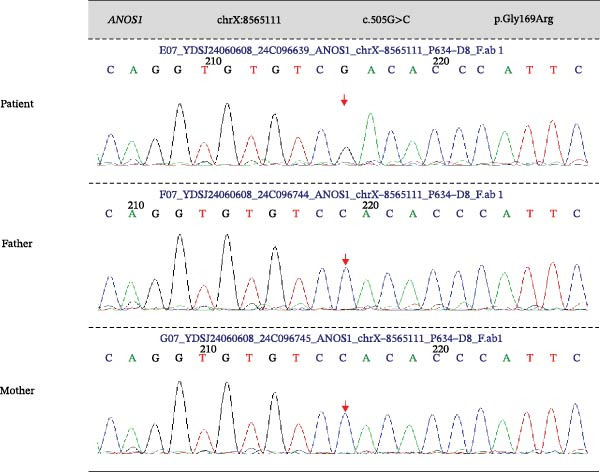
Results of *ANOS1* Sanger sequencing in this proband. Sequencing identified a hemizygous missense variant in the X‐linked *ANOS1* gene: NM_000216.4 (exon 4) c.505G >C;p.(Gly169Arg).

**Figure 4:**
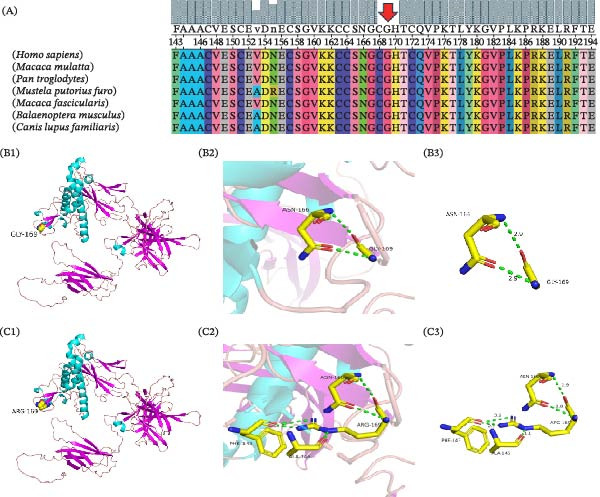
Three‐dimensional structure of wild‐type *ANOS1* and its variant. (A) Cross‐species multiple sequence alignment. (B1, B2, B3) correspond to the wild‐type *ANOS1*; (C1, C2, C3) correspond to the *ANOS1* c.505G>C variant. In the variant, the glycine (Gly) amino acid residue at position 169 was replaced by arginine (Arg), and the variant exhibited an increase of two hydrogen bonds compared with the wild‐type configuration. These additional hydrogen bonds were formed with the side chain of the phenylalanine residue at position 143 and the backbone of the alanine residue at position 146, respectively.

Chromosomal analysis revealed a normal male karyotype (46, XY), along with a chromosomal polymorphism characterized by an elongated satellite stalk on chromosome 21. To identify the genetic variants linked to the phenotypic characteristics of the proband, we conducted trio‐based ES on peripheral blood samples. Molecular analysis detected a novel hemizygous c.505G >C;p.(Gly169Arg) variant in the *ANOS1* gene (NM_000216.4; exon 4; chrX:8597070 G >C; GRCh38/hg38). The variant was confirmed by Sanger sequencing, with representative chromatograms shown in Figure 3. The average read depth was 100× at this locus, and the variant allele fraction was 1.0 (0 reference reads and 50 variant reads), consistent with a hemizygous genotype in the male proband. The relevant descriptions have been updated in Supporting Information 1: Table S2. Notably, this variant was not recorded in the National Center for Biotechnology Information ClinVar database. However, neither parent carried the aforementioned gene variant, confirming a de novo origin of the variant. Biological maternity and paternity were not confirmed. In accordance with the American College of Medical Genetics and Genomics (ACMG) guidelines [16], the PM6 criterion applies to de novo variants for which maternity and paternity have not been verified. Based on the combined evidence (PM2_Supporting + PM6 + PP3_Moderate + PP4), the *ANOS1* c.505G >C;p.(Gly169Arg) variant (transcript NM_000216.4, GRCh38/hg38) is classified as likely pathogenic (Supporting Information 1: Table S2 and Table S3). Multiple sequence alignments showed that the Gly169 amino acid is highly conserved across various species, including *Homo sapiens*, *Macaca mulatta*, *Pan troglodytes*, *Canis lupus familiaris*, and others (Figure 4A). A three‐dimensional protein model illustrated that Gly169 in the wild‐type ANOS1 protein forms two hydrogen bonds; this increases to four when Gly169 is substituted with Arg. Furthermore, additional hydrogen bonds were formed between phenylalanine at position 143 and alanine at position 146. SWISS‐MODEL predicted that the substitution of Gly169 by Arg could significantly alter the conformation of the ANOS1 protein, as indicated in Figure 4B3,C3.

To enhance spermatogenesis and meet the patient’s fertility needs, subcutaneous pulsatile GnRH pump therapy was initiated at a dose of 10 μg per pulse, with pulses being administered at 90 min intervals. Effective glycemic control was achieved through a comprehensive diabetes management plan, including insulin and oral hypoglycemic agents.

Six months after hospital discharge, the patient’s HbA1c level decreased to 6.5%. Concurrently, serum testosterone remained low, with LH and FSH levels recorded at 2.51 and 7.57 IU/mL, respectively. Due to the suboptimal treatment response, the GnRH pump dosage was adjusted from the initial 10 to 15 μg per pulse. Additionally, owing to the sustained effectiveness of glycemic control, insulin was discontinued, and the patient was managed solely with oral hypoglycemic agents (metformin, empagliflozin, and acarbose).

At the fifth follow‐up visit, due to economic considerations, GnRH pump therapy was switched to a combination of hCG and human menopausal gonadotropin. At the sixth follow‐up visit, the patient’s HbA1c level had increased to 6.9%, a level considered consistent with good glycemic control. However, serum testosterone remained low (baseline: 1.21 nmol/L and current: 0.27 nmol/L), while LH and FSH levels were less than 0.3 and 0.57 IU/mL, respectively (compared with baseline levels of 0.38 and 0.86 IU/mL). Given the patient’s persistently low serum testosterone levels and the decision to forgo his fertility needs, testosterone replacement therapy was initiated at a daily dosage of 40 mg to optimize sexual function and metabolic health. At the eighth follow‐up visit (March 8, 2026), serum testosterone was 3.71 nmol/L, and the dose of testosterone undecanoate capsules was adjusted to 80 mg twice daily. The patient’s medical history is described in full chronological sequence, and the timeline of this case is presented in Supporting Information 2: Figure S1.

## 4. Discussion

In this study, we present a case of KS with a novel hemizygous variant in the *ANOS1* gene, c.505G >C;p.(Gly169Arg). Our patient presented with CHH, anosmia, and concurrent T2DM. Diagnostic evaluations, including the GnRH stimulation test, hCG stimulation test, and olfactory test, indicated the presence of KS. Trio‐based ES of the *ANOS1* gene identified a hemizygous variant, c.505G >C;p.(Gly169Arg), which is likely pathogenic for KS and has not been previously reported in the ClinVar database. Notably, neither parent carried the variant, confirming a de novo origin, which further supports its potential pathogenic role. Multiple sequence alignments demonstrated that the Gly169 residue was highly conserved across various species. Furthermore, a three‐dimensional protein model generated by SWISS‐MODEL predicted significant alterations in protein conformation due to the amino acid substitution at this highly conserved site, reinforcing its potential functional significance.

**Figure 5:**
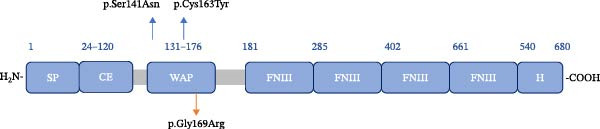
Schematic diagram of the human anosmin‐1 protein structure. Blue arrows indicate previously reported likely‐pathogenic missense amino acid variants, and the red arrow denotes the missense variant identified in the present case.

The *ANOS1* gene, formerly known as *KAL1* (Mendelian Inheritance in Man, MIM: 300,836), was the first gene implicated in X‐linked KS [17]. Pathogenic variants in this gene account for approximately 8% of all KS cases [3], and cryptorchidism is frequently observed. KS patients with *ANOS1* variants frequently exhibit olfactory bulb aplasia or dysplasia [11]. Consistent with this established phenotypic feature of *ANOS1* variants, the patient in our case harbored the aforementioned variant and has confirmed anosmia. Genomically, *ANOS1* is localized to Xp22.31, comprises 14 exons, and exhibits high sequence conservation across species [18]. This gene encodes anosmin‐1, a protein belonging to the fibronectin type III domain superfamily [19]. Anosmin‐1 consists of an N‐terminal signal peptide, a cysteine‐rich region, a whey acidic protein‐like four‐disulfide core motif, four consecutive fibronectin type III domains, and a histidine‐rich C‐terminal segment (Figure 5) [18]. *ANOS1* missense variants located in the potential mutational hotspot spanning amino acids 131–176 were retrieved from the ClinVar database and are summarized in Supporting Information 3: Table S4. The included likely pathogenic missense variants are highlighted in Figure 5 to further verify this potential mutational hotspot domain. In olfactory cells and cerebellar Purkinje neurons, anosmin‐1 promotes axonal outgrowth and acts as an axonal guidance molecule [20]. Notably, GnRH‐producing neurons navigate through the extracellular matrix, utilizing anosmin‐1 as a guide [11]. Previous reports have documented *ANOS1* pathogenic variants including deletions spanning exons 4–14, missense variants, frameshift variants in exon 3, and exon 3 deletions [21, 22], but none have described the c.505G >C;p.(Gly169Arg) variant in exon 4. The novelty of this case lies in the identification of a de novo hemizygous *ANOS1* variant c.505G >C;p.(Gly169Arg) in exon 4, which is predicted to impair protein function and contribute to KS pathogenesis.

DM is a potential comorbidity in patients with KS as the disease progresses, and androgens play a key role in maintaining glucose homeostasis. Specifically, androgens facilitate glucose uptake, upregulate the expression of glucose transporter (GLUT) to enhance glucose transport, and activate the adenosine 5′‐monophosphate‐activated protein kinase (AMPK) signaling pathway to promote glucose consumption [23, 24]. Long‐standing hypogonadism leads to insulin resistance, dyslipidemia, reduced lean body mass, decreased bone mineral density, and increased adiposity [25–27]. A clear association exists between reduced total testosterone levels and T2DM [28]. Although testosterone treatment may prevent progression from impaired glucose tolerance to T2DM, insufficient safety data are available to support its use for diabetes prevention [28]. Notably, variants in genes including *KLB* (p.Thr313Met), *IGSF10* (p.Lys1819Arg and p.Arg1035Thr), and *ANOS1* (p.Cys172Phe) may be associated with the pathogenesis of DM [11]. However, a single case of familial diabetes carrying the *ANOS1* variant is insufficient to establish a definitive causal relationship. By 2040, T2DM will affect approximately 642 million people worldwide [29], and age, male sex, hypertension, cigarette smoking, obesity, and a family history of diabetes are significant risk factors for T2DM [30]. In this patient, a family history of diabetes, lifestyle factors, obesity, or other genetic risk factors may also contribute to the occurrence of T2DM, independent of the *ANOS1* variant. In contrast, the evidence supporting a direct effect of the *ANOS1* gene on glucose metabolism remains limited. Owing to the small sample size and limitations of published studies, larger genotype–phenotype investigations and functional studies are required before any definitive association can be confirmed. Consistent with this, the *ANOS1* p.Gly169Arg variant identified in our 33‐year‐old male patient is also associated with T2DM, and bioinformatics predictions (SIFT, PolyPhen‐2, and MutationTaster) suggest that this variant is likely deleterious. As these predictions are only for hypothesis generation, the functional impact remains unproven in the absence of biochemical or cellular assays, and further mechanistic studies are warranted to validate its biological effects. Meanwhile, the precise mechanism by which the *ANOS1* p.Gly169Arg variant contributes to T2DM in this patient remains unclear, necessitating further in vitro and in vivo experimental validation.

## 5. Conclusions

In conclusion, incomplete development of secondary sexual characteristics, combined with hyposmia/anosmia and hypogonadotropic hypogonadism, should raise clinical suspicion for KS, warranting prompt genetic testing to confirm the diagnosis. Using ES, we identified a novel *ANOS1* variant that is likely pathogenic for KS and may contribute to T2DM in a young male patient. Our findings suggest that *ANOS1* may act as a potential cofactor implicated in both KS and T2DM. This finding highlights the utility of genetic testing (particularly ES) in confirming KS diagnoses and identifying novel pathogenic variants, supporting its role in clinical practice. To advance our understanding of the interplay between KS and T2DM, functional studies (e.g., in vitro protein assays and animal models) are needed to clarify how this variant impacts ANOS1 function and its role in both KS pathogenesis and glucose metabolism.

## Author Contributions

Si Qin, Yinxing Ni, and Jian Zhong were involved in medical practices. Si Qin, Yinxing Ni, and Jian Zhong were involved in concept and design. Si Qin, Yindi Zhang, and Yanrong Chen were involved in data collection or processing. Si Qin, Yindi Zhang, and Wei Chen were involved in analysis or interpretation and literature search. Si Qin, Yinxing Ni, and Jian Zhong were involved in writing and wrote the main manuscript text. Yindi Zhang, Wei Chen, and Yanrong Chen prepared Figures 1–5.

## Funding

No funding was received for this manuscript.

## Disclosure

All the authors reviewed the manuscript. All authors have read and approved the final version of the manuscript. Jian Zhong had full access to all of the data in this study and takes complete responsibility for the integrity of the data and the accuracy of the data analysis.

## Ethics Statement

The study was conducted in accordance with the World Medical Association Declaration of Helsinki and with the written informed consent of this participant. Furthermore, the study protocol was approved by our Institutional Review Board (Research Project Number 20240064).

## Consent

The patient provided written informed consent for the study. The authors affirm that human research participants provided informed consent for publication of all data reported in this study, including the images in Figures 1–5.

## Conflicts of Interest

The authors declare no conflicts of interest.

## Supporting Information

Additional supporting information can be found online in the Supporting Information section.

## Supporting information


**Supporting Information 1** TABLE S1: Pathogenicity prediction tools. TABLE S2: Variant curation. TABLE S3: Variant curation using the specific American College of Medical Genetics and Genomics evidence codes.

**Supporting Information 2** Figure S1: The timeline of this case.

**Supporting Information 3** TABLE S4: Summary of reported ANOS1 missense variants clustered in the mutational‐hotspot region (amino acids 131–176) retrieved from the ClinVar database.

## Data Availability

The data that support the findings of this study are available from the corresponding author upon reasonable request.
