# Supplementary material for: A Novel Hemizygous ANOS1 Variant in a Patient With Kallmann Syndrome and Type 2 Diabetes Mellitus: A Case Report
Source: Case Rep Endocrinol. 2026 Jul 22;2026:8624241. doi: 10.1155/crie/8624241 (PMC13392514; doi:10.1155/crie/8624241)
Supplement: Supplementary file 1 — Supporting Information 1 TABLE S1: Pathogenicity prediction tools. TABLE S2: Variant curation. TABLE S3: Variant curation using the specific American College of Medical Genetics and Genomics evidence codes. [file CRIE-2026-8624241-s002.doc]

Supplementary TABLE 1 Pathogenicity prediction tools

| **Prediction tool** | **Version** | **Score** | **Threshold** |
| --- | --- | --- | --- |
| SIFT | SIFT ensembl 66, released Jan, 2015 | 0.004 | The SIFT score ranges from 0 to 1.  SIFT score < 0.05, Deleterious  SIFT score ≥ 0.05, Tolerated |
| MutationTaster | MutationTaster2025 | D | A: Disease causing automatic  D: Disease causing  N: Polymorphism  P: Polymorphism automatic |
| PolyPhen-2 | v2.2.2 | 1 | The PolyPhen-2 score ranges from 0 to 1.  D: Probably damaging: ≥ 0.909  P: Possibly damaging: 0.447–0.909  B: Benign: ≤ 0.446 |
| GERP++ | dbNSFP4.2a | 4.51 | Rejected Substitutions (ES) > 2 suggests strong evolutionary constraint and functional importance. |
| REVEL | v1.3 | 0.864 | 1. Pathogenic (PP3)  1.1 Very Strong -  1.2 Strong ≥ 0.932  1.3 Moderate [0.7773, 0.932)  1.4 Supporting [0.644, 0.7773)  2.Benign (BP4)  2.1 Supporting (0.183, 0.290]  2.2 Moderate (0.016, 0.183]  2.3 Strong (0.0003, 0.016]  2.4 Very Strong ≤ 0.0003 |

SIFT, Sorting Intolerant From Tolerant; PolyPhen-2, Polymorphism Phenotyping v2; GERP++, Genomic Evolutionary Rate Profiling++; REVEL, Rare Exome Variant Ensemble Learner.

Supplementary TABLE 2 Variant curation.

|  | **Gene** | **Genomic coordinates, GRCh38 (hg38)** | **Transcript** | **Exon** | **Mutation** | **Variant segregation** | **Normal frequency**  **(****1000g2015aug_all,** **gnomAD_exome_ALL, and gnomAD_exome_EAS)** | **Read depth** | **Allele balance** | **ClinVar status** | **ACMG Pathogenicity Analysis** | |
| --- | --- | --- | --- | --- | --- | --- | --- | --- | --- | --- | --- | --- |
| *ANOS1* | | chrX:8597070 | NM_000216.4 | 4 | c.505G>C (p.Gly169Arg) | Hemizygous (hemi) | - | 100× | 50/0 (100% mutant allele, hemizygous on chrX) | No reported | Likely Pathogenic |  |

GRCh37/hg19, Genome Reference Consortium Human Build 37/human genome 19; 1000g2015aug_all, 1000 Genomes Project 2015 August overall population; gnomAD, Genome Aggregation Database; gnomAD_exome_ALL, gnomAD exome overall population; gnomAD_exome_EAS, gnomAD exome East Asian population; gnomAD, Genome Aggregation Database; ClinVar, Clinical Variation Database; ACMG, American College of Medical Genetics and Genomics; chrX, X chromosome.

Supplementary TABLE 3 Variant curation using the specific American College of Medical Genetics and Genomics evidence codes.

| **ACMG Code** | **Rationale** |
| --- | --- |
| **PM2_Supporting** | The frequency in the normal population database is “–”. |
| **PM6** | Spontaneous mutation, but parental origin is uncertain or the clinical phenotype is poorly consistent. |
| **PP3_Moderate** | The REVEL software, a comprehensive protein function prediction tool, predicts this as harmful. |
| **PP4** | Diseases with highly specific phenotypes or family histories attributable to a single genetic basis. |
