# Supplementary material for: A Novel Hemizygous ANOS1 Variant in a Patient With Kallmann Syndrome and Type 2 Diabetes Mellitus: A Case Report
Source: Case Rep Endocrinol. 2026 Jul 22;2026:8624241. doi: 10.1155/crie/8624241 (PMC13392514; doi:10.1155/crie/8624241)
Supplement: Supplementary file 2 — Supporting Information 2 Figure S1: The timeline of this case. [file CRIE-2026-8624241-s001.docx]

Insulin and oral hypoglycemic agents

Insulin was discontinued

GnRH pump therapy

Combination of HCG and HMG

The 7th follow-up

The 5th follow-up

June 5^th^

MRI testing

Testosterone replacement therapy, 40 mg/day

Testosterone replacement therapy, 80 mg twice daily

The 8th follow-up

2026

March 8^th^

October 19^th^

August 9^th^

2025

May 17^th^

The 6th follow-up

The first follow-up

August 26^th^

May 29^st^

Trio-based whole-exome sequencing

Discharge from hospital

May 27^th^

May 23^th^

May 19^th^

May 22^th^

Plasma glucose and C-peptide responses to oral glucose tolerance test

Olfactory testing

May 2024

HCG stimulation tests

GnRH stimulation tests

**33-year-old male**

**evaluated for newly diagnosed hyperglycemia in May 17 2024**
